# The social determinants of tuberculosis: a case-control study characterising pathways to equitable intervention in Peru

**DOI:** 10.1186/s40249-025-01324-6

**Published:** 2025-06-20

**Authors:** Matthew J. Saunders, Rosario Montoya, Luz Quevedo, Eric Ramos, Sumona Datta, Carlton A. Evans

**Affiliations:** 1 Institute for Infection and Immunity, City St. George’s, University of London, London, UK; 2 Faculty of Public Health and Policy, London School of Hygiene & Tropical Medicine, London, UK; 3 Innovation for Health and Development (IFHAD), Laboratory of Research and Development, Faculty of Sciences and Engineering, Universidad Peruana Cayetano Heredia, Lima, Perú; 4 Innovación Por La Salud Y Desarrollo (IPSYD), Asociación Benéfica PRISMA, Lima, Perú; 5 Innovation for Health and Development (IFHAD), Department of Infectious Disease, Imperial College London, London, UK

**Keywords:** Tuberculosis, Social determinant, Risk factor, Poverty, Peru

## Abstract

**Background:**

Despite being key components of global tuberculosis policy, poverty reduction and social protection interventions have been inconsistently implemented. We aimed to characterise how poverty and interrelated personal risk factors increase tuberculosis risk in Peru to inform the design of locally appropriate, person-centred, equity-oriented interventions.

**Methods:**

We undertook a case-control study among people aged 15 years and over in 32 communities in Peru between 2016 and 2019. Cases (*n* = 2337) were people diagnosed with any form of tuberculosis. Controls (*n* = 981) were people living in randomly selected households in the same communities. We derived measures of household poverty from three dimensions (physical, human, and financial capital) and investigated the associations between these; personal risk factors more specifically linked to health (e.g. smoking); and tuberculosis. We used logistic regression to calculate adjusted odds ratios (a*OR*), 95% confidence intervals (95% *CI*), and population attributable fractions (PAF). A directed acyclic graph was used to inform the analytical approach.

**Results:**

Household poverty was strongly associated with tuberculosis (a*OR* = 3.1; 95% *CI:* 2.3–4.2 for people from the ‘poorer’ versus ‘less poor’ half of households). There was a non-linear social gradient across deciles of household poverty, with odds of tuberculosis increasing exponentially as poverty deepened (a*OR* = 12.6; 95% *CI:* 6.8–23.2 for the ‘poorest’ decile versus the ‘least poor’ decile). Overall, tuberculosis burden could be halved by reducing poverty in the ‘poorer’ half of households to the level of the ‘less poor’ half (PAF = 47%; 95% *CI:* 40–54). For key personal risk factors, we estimated PAF for alcohol excess (PAF = 12.3%, 95% *CI:* 7.2–17.2); underweight (PAF = 10.3%, 95% *CI:* 8.7–11.8); smoking (PAF = 8.8%, 95% *CI:* 3.8–13.5); HIV (PAF = 5.7%, 95% *CI:* 4.6–6.7); and diabetes (PAF = 4.6%, 95% *CI:* 3.3–6.0). We also identified other important risk factors including previous tuberculosis (PAF = 14.8%, 95% *CI:* 11.6–17.9); incarceration (PAF = 9.5%, 95% *CI:* 6.8–12.1); and lower social capital (PAF = 4.1%, 95% *CI:* 2.6–5.6). Most personal risk factors, particularly education and substance misuse, tuberculosis exposures (e.g. incarceration and homelessness), and undernutrition, exhibited a social gradient across quintiles of household poverty and were more prevalent in people living in poorer households (Cochran-Armitage test for linear trend *P* < 0.001 for variables showing these social gradients).

**Conclusions:**

Interventions addressing multidimensional household poverty and interrelated personal risk factors could substantially reduce tuberculosis burden. Our results provide an evidence base for designing person-centred, equity-oriented interventions; and support more effective implementation of poverty reduction and social protection within the global tuberculosis response.

**Supplementary Information:**

The online version contains supplementary material available at 10.1186/s40249-025-01324-6.

## Background

The global tuberculosis epidemic is driven by the social determinants of health – the conditions in which people are born, grow, work, live and age and the wider set of economic and political forces shaping daily life [1, 2]. This is starkly illustrated by the reductions in tuberculosis observed in Europe and North America in the late 19th and early twentieth centuries, which have been largely attributed to socioeconomic development [3]. More recently, several studies have demonstrated how tuberculosis rates change in association with national poverty indicators and social protection spending [4–6]; and characterised the association between specific socially determined risk factors, such as undernutrition, and tuberculosis risk [7, 8].

The World Health Organization (WHO) ‘End TB Strategy’ and United Nations (UN) Sustainable Development Goals (SDGs) recognise this and explicitly conceptualise tuberculosis as a development challenge and opportunity [9, 10]. The End TB Strategy mandates that zero tuberculosis-affected households face catastrophic costs[11] by 2030 and calls for the expansion of poverty reduction interventions (aiming to create sustainable pathways out of poverty) and social protection interventions (aiming to reduce vulnerability to poverty) for people and communities at high risk of tuberculosis. Supporting this, modelling has suggested that full achievement of SDG 1 (ending extreme poverty and expanding social protection) could reduce tuberculosis incidence by between 55% and 95% [12].

To date, however, many interventions of this nature implemented for tuberculosis have focussed narrowly on providing cash transfers to people already affected by tuberculosis – termed tuberculosis-specific social protection [13]. Broader poverty reduction and social protection interventions aiming to reduce tuberculosis risk (termed tuberculosis-sensitive interventions) may be more complex to implement, requiring multisectoral collaboration and sustained financing. However, the impact of COVID-19 has demonstrated the importance of combining disease-specific programmes with those that address social determinants and underlying vulnerabilities [14]. Investing in addressing all aspects of poverty may not be feasible in the context of tuberculosis prevention [15]. Thus, to inform the design of poverty reduction and social protection interventions that are locally appropriate, person-centred, and equity-oriented, data are needed to understand how social determinants influence tuberculosis risk and to characterise causal mechanisms.

In this study, we aimed to characterise how poverty (principally conceptualised and measured at the household level) and interrelated personal risk factors more specifically linked to health (e.g. smoking) increase tuberculosis risk in Peru.

## Methods

### Study design and setting

This was a case-control study nested within the “PREVENT TB” study, undertaken in Callao, Peru, which borders the capital Lima and is part of its metropolitan area [16]. We have worked with 32 of the 45 communities constituting Callao since 2013, selected for their high tuberculosis rates. Each community is served by a Ministry of Health (MINSA)-run health post providing their population with primary care, including tuberculosis services. In 2019, approximately 900,000 people lived in these 32 communities and the tuberculosis case notification rate collected collaboratively with MINSA-run health posts was 135/100,000 people. Our study was undertaken with the approval and collaboration of the Peruvian National Tuberculosis Programme and participating health posts. Ethical approvals included the Callao Ministry of Health, Peru; Asociación Benéfica PRISMA, Peru; and Imperial College London, UK.

### Participants

Cases were people diagnosed with tuberculosis (pulmonary or extra-pulmonary, with or without bacteriological confirmation, i.e. diagnosed on the basis of symptoms or radiological changes), principally through passive case finding, who were awaiting or receiving treatment at MINSA-run health posts in participating communities [17]. Study research nurses worked in collaboration with these health posts to invite cases to participate as soon as they were diagnosed. If a case had two episodes of tuberculosis during the study period, they were invited to participate twice. If two people in the same household were diagnosed with tuberculosis, both were invited to participate.

Controls were people living in randomly selected households in participating communities on the premise that, if they were diagnosed with tuberculosis, they would be offered treatment in the same MINSA-run health posts as the cases. To select controls, residential blocks were first enumerated using a satellite map and then randomly selected using random number tables. Then, using another random number table, a residential property within the block was randomly selected. The North-West corner of the block was located and from there residential properties were counted in a clockwise direction and the household corresponding to the random number selection was approached. If no adults (people aged 18 years or over) were available or willing to provide informed written consent to participate, another randomly selected property in the selected block was visited. When an adult provided consent, all other household members were also invited to participate. Controls who had symptoms of tuberculosis were referred to local healthcare services. If they were then diagnosed or self-reported having tuberculosis at the time of recruitment, they were reclassified as cases.

All cases and controls who were willing and able to give their written informed consent and, in the case of minors (aged under 18 years), assent, were eligible to be recruited to the PREVENT TB study. The current study was restricted to people aged 15 years and over with data available for analysis because of differences in tuberculosis epidemiology, diagnosis, and risk factors in children. The number of cases included was defined by the sample size calculation for the PREVENT TB study. The number of controls aimed to total half the number of cases and was calculated in proportion to the population size of each community to ensure a sample representative of the underlying population. Recruitment took place between July 2016 and April 2019.

### Data collection and theoretical framework

**Fig. 1:**
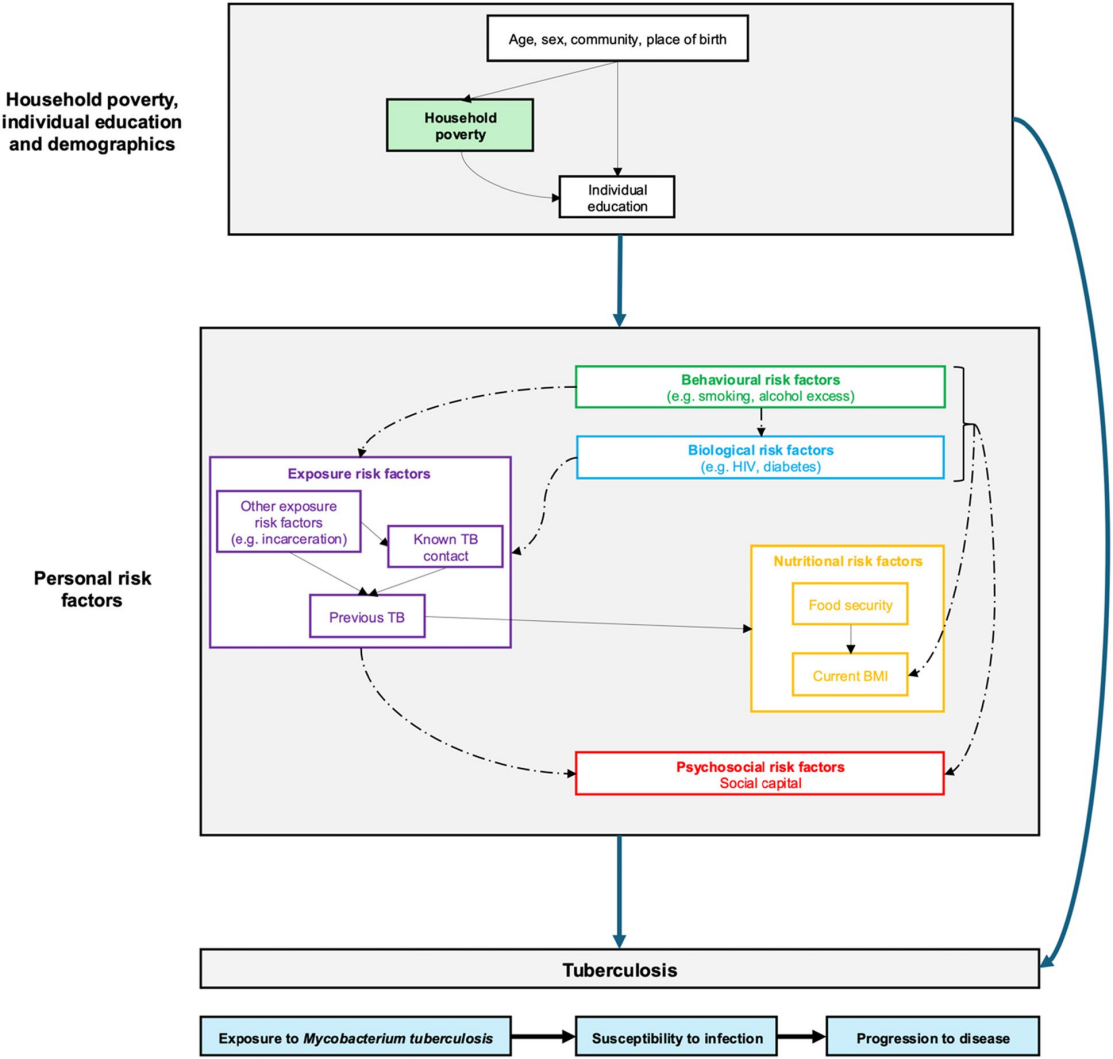
Simplified directed acyclic graph illustrating hypothesised causal relationships between household poverty, personal risk factors, and tuberculosis. *TB* tuberculosis; *BMI* body mass index. Solid arrows indicate that all variables in the upstream node (illustrated as boxes) were hypothesised to cause all variables in the downstream node. Dashed arrows indicate that only some variables in the upstream node were hypothesised to cause some variables in the downstream node, e.g. biological risk factors were hypothesised to cause hospitalisation and previous TB in the exposure node, but not the other exposure risk factors. See Table 2 for all variables under study

This study was undertaken explicitly from social epidemiologic perspectives of disease distribution [18]. A directed acyclic graph (DAG), informed by a literature review, was drawn to illustrate the pathways through which we hypothesised household poverty and interrelated downstream personal risk factors to be causally associated with tuberculosis [19]. A simplified version of this DAG is shown in Fig. 1 and further information on our approach can be found in the Supplementary Appendix (page 1).

All questionnaires were refined through extensive pilot work. All cases and the first consenting adult from each control household completed a questionnaire with a trained research nurse to characterise three broad dimensions of household poverty based on the capital assets outlined in the Sustainable Livelihood Framework [20].

- *Physical capital* measured household crowding, building ownership and quality, access to basic services (e.g. sanitation), and asset ownership (e.g. television).
- *Human capital* measured education of the heads of the household and internet usage in the last week as a proxy for knowledge, skills, and digital literacy.
- *Financial capital* measured household income (pre-illness for cases) and food spending per capita, food availability, savings, debt, and bank account ownership.

Additionally, all cases and controls completed a questionnaire to characterise personal risk factors, which were broadly categorised into five domains.

- *Education and behavioural* risk factors included the individual’s education level, and whether they had previously regularly smoked, drank alcohol to excess (both subjectively interpreted by each participant), or used other drugs common in this setting (e.g. a precursor to cocaine).
- *Exposure* risk factors included known risk factors for tuberculosis exposure, such as incarceration and having ever lived with someone with tuberculosis.
- *Biological* risk factors included Bacillus Calmette-Guérin (BCG) vaccination, assessed through visualising the BCG scar; and self-reported prevalence of diabetes, HIV, or other known immunosuppression (either from daily corticosteroid usage or other chronic diseases).
- *Nutritional* risk factors included underweight [defined as a body mass index (BMI) measured by research nurses as < 18.5 kg/m^2^ for people aged 18 years and over and as WHO BMI-for-age Z score < −2 for people aged 15–17 years] and food insecurity (defined as going to bed hungry because of lack of food on at least one day in the last month).
- *Psychosocial* risk factors included the individual’s perceived social capital over the last 12 months, measured using an adapted version of the Short Adapted Social Capital Assessment Tool to generate a continuous social capital score (SASCAT) [21].

To ensure quality control, questionnaires were digitised into a database with field limits and validation rules and where missing data or implausible values were noted, questionnaires were returned to the research nurse for correction with the study participant.

### Statistical analysis

All analyses were performed using Stata version 18 (StataCorp, Texas, USA) and RStudio version 2023.03.1 + 446 (Lucent Technologies, Jasmine Mountain, USA). Continuous variables were summarised by their means and standard deviations (*SD*) or medians and interquartile ranges (*IQR*). Binary and categorical variables were summarised as proportions. Although the proportion of participants with missing data was small for most variables (< 1%), we used multiple imputation with chained equations to replace missing values (Supplementary Appendix, pages 2–4).

We used logistic regression to investigate the associations between household poverty, personal risk factors and tuberculosis, adjusting for household clustering (because there were multiple controls per household) by calculating robust standard errors. We first investigated the independent associations between three discrete measures of household poverty and tuberculosis:

- crowding (number of people sleeping per room as an ordinal variable) as a measure of physical capital [20];
- education level of the female head of the household (as an ordinal variable) as a measure of human capital [20]; and
- monetary poverty as a measure of financial capital, defined as a dichotomous variable using the Peruvian poverty line based on household income per capita [22].

We calculated odds ratios (a*OR*) with 95% confidence intervals (95% *CI*) for different levels of these variables compared to the reference category, adjusted for each other and for age, sex, community, and place of birth. Then, we dichotomised crowding and female education at the point where the odds of tuberculosis increased and calculated the population attributable fraction (PAF) of tuberculosis due to each of the three measures [23].

We then used principal components analysis (PCA) of all 27 household poverty variables to derive a continuous index of overall household poverty following published guidance [24]. To derive an index that represented the underlying distribution of poverty in Callao, PCA was undertaken only in the control households and then the weights for each of the variables from the first principal component were applied to all households. We dichotomised this index at the control household median value into ‘poorer’ versus ‘less poor’ households to provide a clearly interpretable comparison; investigated its association with tuberculosis adjusting for age, sex, community, and place of birth; and calculated the PAF to estimate the reduction in tuberculosis burden that would occur if the poorer households in Callao experience a reduction in poverty to the level of the less poor half of the population. Then, to investigate the relative independent importance of each of the dimensions of household poverty, we derived separate PCA indices for each dimension and calculated their a*OR* and 95% *CI* for tuberculosis, adjusted for each other and for age, sex, community, and place of birth. We also investigated whether there was a social gradient in tuberculosis by deriving deciles of household poverty and calculating the a*OR* and 95% *CI* for tuberculosis in each decile compared to the least poor decile.

Finally, we investigated the associations between each personal risk factor and tuberculosis and calculated PAF. Rather than building a single multivariable model including all risk factors, we built separate models for each risk factor with adjustment sets for each based on our DAG [25]. To illustrate the interrelationships between household poverty and these personal risk factors and provide insight into the individual-level pathways through which household poverty may increase tuberculosis risk, we plotted the prevalence of these risk factors across quintiles of household poverty and compared them using the Cochran-Armitage test for linear trend. Because we aimed to quantify the individual contributions of different risk factors, we restricted our analyses to main effects without investigating the multiple interactions possible.

## Results

### Recruitment

A total of 2785 cases were identified, of whom 2484 (89%) were recruited and 2346 were aged over 15 years. For controls, 81% (1407/1745) people in 378 households were recruited and 986 were aged over 15 years. After reclassifying two controls who had current tuberculosis to become cases and excluding one control who had already been recruited as a case and 13 people who had no data available, there were 2337 cases and 981 controls. Among cases, the median age was 31 years (*IQR* = 23–47) and 64% (*n* = 1499) were male. Among controls, the median age was 38 years (*IQR* = 25–54) and 40% (*n* = 389) were male.

### Household poverty and tuberculosis

**Fig. 2:**
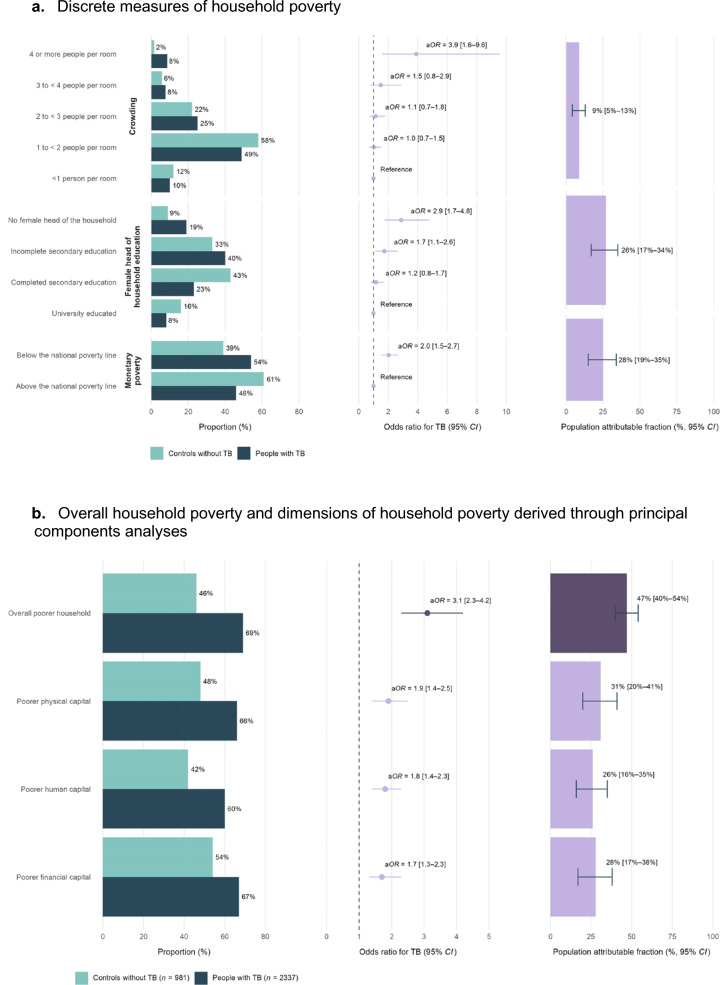
Associations between household poverty and tuberculosis (TB) (*n* = 3318). **a** Discrete measures of household poverty. **b** Overall household poverty and dimensions of household poverty derived through principal components analyses. Odds ratios (a*OR*) and population attributable fractions (PAF) were adjusted for age, sex, community, and place of birth for all variables shown here. For discrete measures of household poverty, a*OR* and PAF were also adjusted for the other variables in the figure. For physical, human, and financial capital, a*OR* and PAF were also adjusted for the other dimensions. Error bars represent 95% confidence intervals (95% *CI*). Numbers and p values are shown in the Supplementary Appendix, page 5

**Fig. 3:**
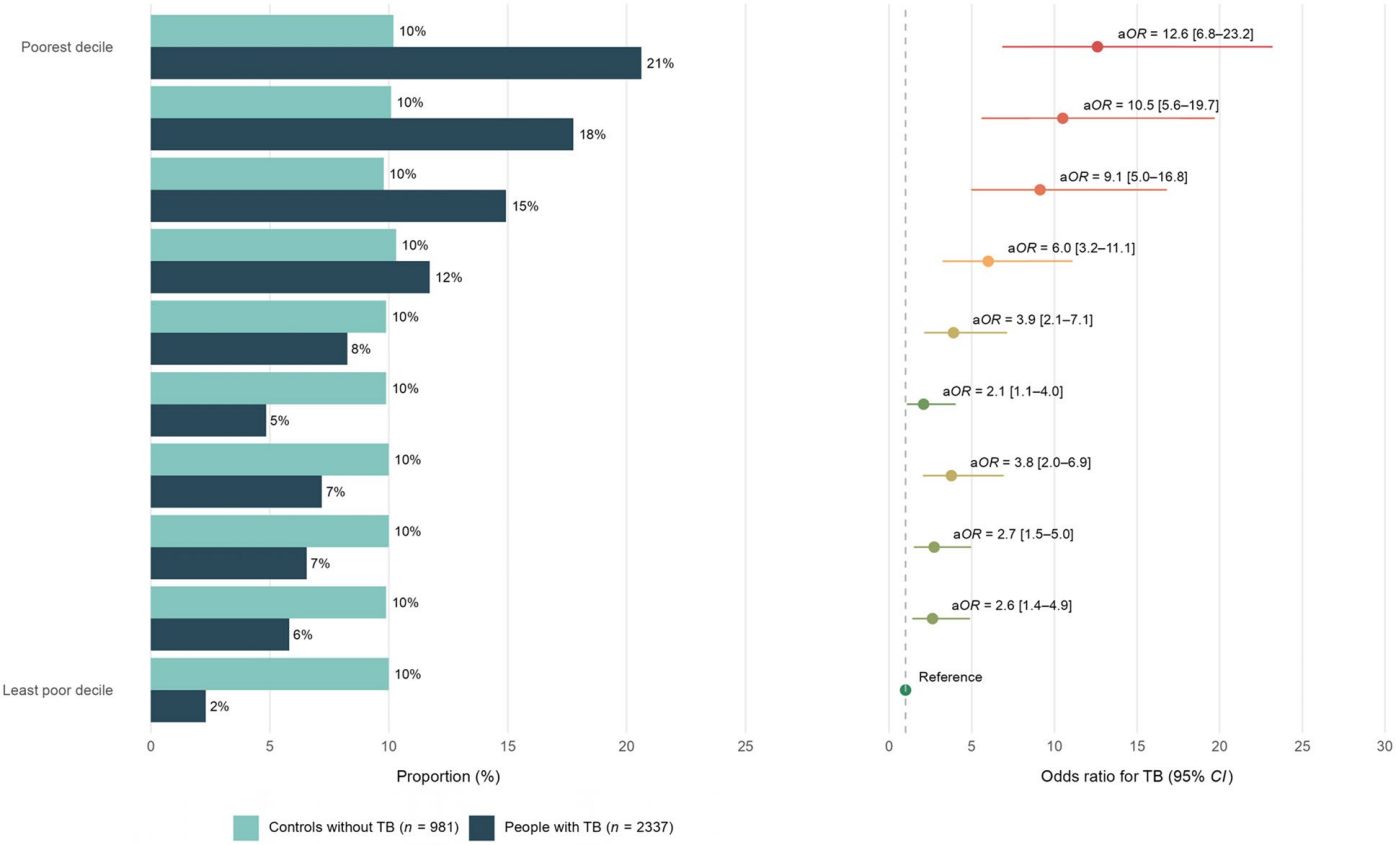
Social gradient in tuberculosis (TB) across deciles of household poverty (*n* = 3318). Odds ratios (a*OR*) were adjusted for age, sex, community, and place of birth. Error bars represent 95% confidence intervals (95% *CI*). Numbers and *P* values are shown in the Supplementary Appendix, page 6

Household poverty variables with weightings derived from PCA, for controls versus cases with tuberculosis, are shown in Table 1. In the analysis of discrete measures of household poverty; crowding, education level of the female head of the household, and monetary poverty were all independently associated with tuberculosis (Fig. 2a and Supplementary Appendix page 5). The PAF indicate that tuberculosis burden could be reduced by 9% (95% *CI*: 5–13) by reducing crowding to less than three people per room; 26% (95% *CI:* 17–34) if all female heads of households complete secondary education; and 28% (95% *CI:* 19–35) if no households live below the Peruvian monetary poverty line. In the analysis using PCA-derived indices; overall household poverty was strongly associated with tuberculosis (a*OR* = 3.1; 95% *CI:* 2.3–4.2 for people from ‘poorer’ versus ‘less poor’ households) and all three dimensions of household poverty (physical, human, and financial capital) were independently associated with tuberculosis to a similar extent (Fig. 2b and Supplementary Appendix page 5). The PAF indicates that tuberculosis burden could be reduced by 47% (95% *CI*: 40–54) if the poorer half of households in Callao experience a reduction in poverty to the level of the less poor half of the population. There was a non-linear social gradient in tuberculosis, with odds increasing exponentially as poverty deepened (Fig. 3 and Supplementary Appendix page 6). 21% of cases were in the poorest decile of household poverty, compared with only 2% in the least poor decile.

**Table 1:** Household poverty variables, with weightings used to create indices of household poverty derived from principal components analysis for controls versus cases with tuberculosis (*n* = 2713)

		Households of people without TB (controls, *n* = 378)	Households of people with TB (cases, *n* = 2335)*	Weighting in overall household poverty index	Weighting in dimension-specific household poverty index
Dimension: Physical capital					
Crowding	4 or more people per room	6 (1.6%)	195 (8.5%)	0.15	0.17
	3 to < 4 people per room	19 (5.2%)	175 (7.7%)		
	2 to < 3 people per room	79 (21.4%)	569 (24.9%)		
	1 to < 2 people per room	204 (55.3%)	1111 (48.6%)		
	< 1 person per room	61 (16.5%)	234 (10.3%)		
Home ownership	No	113 (30.0%)	985 (42.4%)	0.095	0.12
	Yes	264 (70.0%)	1340 (57.6%)		
Wall quality	Low (e.g. adobe)	16 (4.2%)	163 (7.0%)	0.24	0.27
	Medium (e.g. wood)	99 (26.3%)	640 (27.4%)		
	High (e.g. cement)	262 (69.5%)	1530 (65.6%)		
Floor quality	Low (e.g. dirt)	22 (5.8%)	203 (8.7%)	0.27	0.3
	Medium (e.g. basic wood)	240 (63.7%)	1652 (70.9%)		
	High (e.g. tiles)	115 (30.5%)	474 (20.4%)		
Water supply	None	7 (1.9%)	72 (3.1%)	0.17	0.22
	Intermediate (tank or well)	29 (7.7%)	180 (7.7%)		
	Optimal (piped)	340 (90.4%)	2079 (89.2%)		
Toilet	None	5 (1.3%)	45 (1.9%)	0.16	0.20
	Intermediate (latrine)	36 (9.6%)	284 (12.2%)		
	Optimal (piped)	336 (89.1%)	2001 (85.9%)		
Electricity	No	5 (1.3%)	33 (1.4%)	0.14	0.17
	Yes	372 (98.7%)	2298 (98.6%)		
Cooking fuel	Dirtier (e.g. kerosene)	2 (0.6%)	37 (1.7%)	0.063	0.073
	Cleaner (e.g. gas)	358 (99.4%)	2138 (98.3%)		
Television ownership	None	19 (5.1%)	196 (8.4%)	0.28	0.33
	One	164 (43.6%)	1148 (49.4%)		
	Two or more	193 (51.3%)	980 (42.2%)		
Fridge ownership	No	53 (14.1%)	615 (26.4%)	0.27	0.32
	Yes	324 (85.9%)	1717 (73.6%)		
Iron ownership	No	75 (20.2%)	783 (33.8%)	0.3	0.33
	Yes	300 (79.8%)	1535 (66.2%)		
Stove ownership	No	12 (3.2%)	194 (8.3%)	0.094	0.13
	Yes	365 (96.8%)	2137 (91.7%)		
Mobile phone ownership	No	31 (8.2%)	202 (8.7%)	0.16	0.15
	Yes	346 (91.8%)	2127 (91.3%)		
Landline ownership	No	230 (61.0%)	1692 (72.6%)	0.28	0.32
	Yes	147 (39.0%)	640 (27.4%)		
Radio ownership	No	105 (27.9%)	835 (35.8%)	0.17	0.19
	Yes	272 (72.2%)	1495 (64.2%)		
Coffee maker ownership	No	287 (76.3%)	2013 (86.6%)	0.2	0.23
	Yes	89 (23.7%)	311 (13.4%)		
Wardrobe ownership	No	62 (16.5%)	569 (24.4%)	0.17	0.2
	Yes	315 (83.6%)	1760 (75.6%)		
Food processor ownership	No	69 (18.3%)	620 (26.7%)	0.25	0.29
	Yes	308 (81.7%)	1702 (73.3%)		
Dimension: Human capital					
Female head of household education	No female head	41 (11.0%)	427 (19.4%)	0.2	0.56
	Not completed secondary	114 (30.7%)	882 (40.1%)		
	Completed secondary	155 (41.7%)	713 (32.4%)		
	Completed higher	62 (16.7%)	178 (8.1%)		
Male head of household education	No male head	46 (12.5%)	323 (14.5%)	0.18	0.58
	Not completed secondary	99 (26.8%)	732 (33.0%)		
	Completed secondary	157 (42.6%)	909 (40.9%)		
	Completed higher	67 (18.2%)	257 (11.6%)		
Used internet in last week	No	162 (43.2%)	1196 (51.7%)	0.22	0.59
	Yes	213 (56.8%)	1116 (48.3%)		
Dimension: Financial capital					
Household income per month per person	Below the national poverty line	67 (40.1%)	1177 (54.1%)	0.23	0.6
	Above the national poverty line	100 (59.9%)	998 (45.9%)		
Food spending per week person (PEN)	Median (*IQR*)	44 (35–60)	42 (29–58)	0.049	0.5
Number of days of food available	Median (*IQR*)	1 (0–3)	1 (0–2)	0.04	0.16
Any savings	No	315 (92.1%)	2008 (92.4%)	0.062	0.43
	Yes	27 (7.9%)	166 (7.6%)		
Any debt	Yes	119 (34.1%)	888 (40.6%)	0.047	0.15
	No	230 (65.9%)	1302 (59.5%)		
Bank account ownership	No	185 (56.2%)	1285 (68.3%)	0.24	0.39
	Yes	144 (43.8%)	597 (31.7%)		

Data are *n* (%) unless otherwise stated. For percentages, the denominator is considered as households with data available

*TB* tuberculosis; *PEN* Peruvian Nuevos Soles; *SD* standard deviation; *IQR* interquartile range

^*^Two members of control households were reclassified as cases, and their actual household poverty variables were used from the control household from which they had been recruited

### Personal risk factors and tuberculosis

**Fig. 4:**
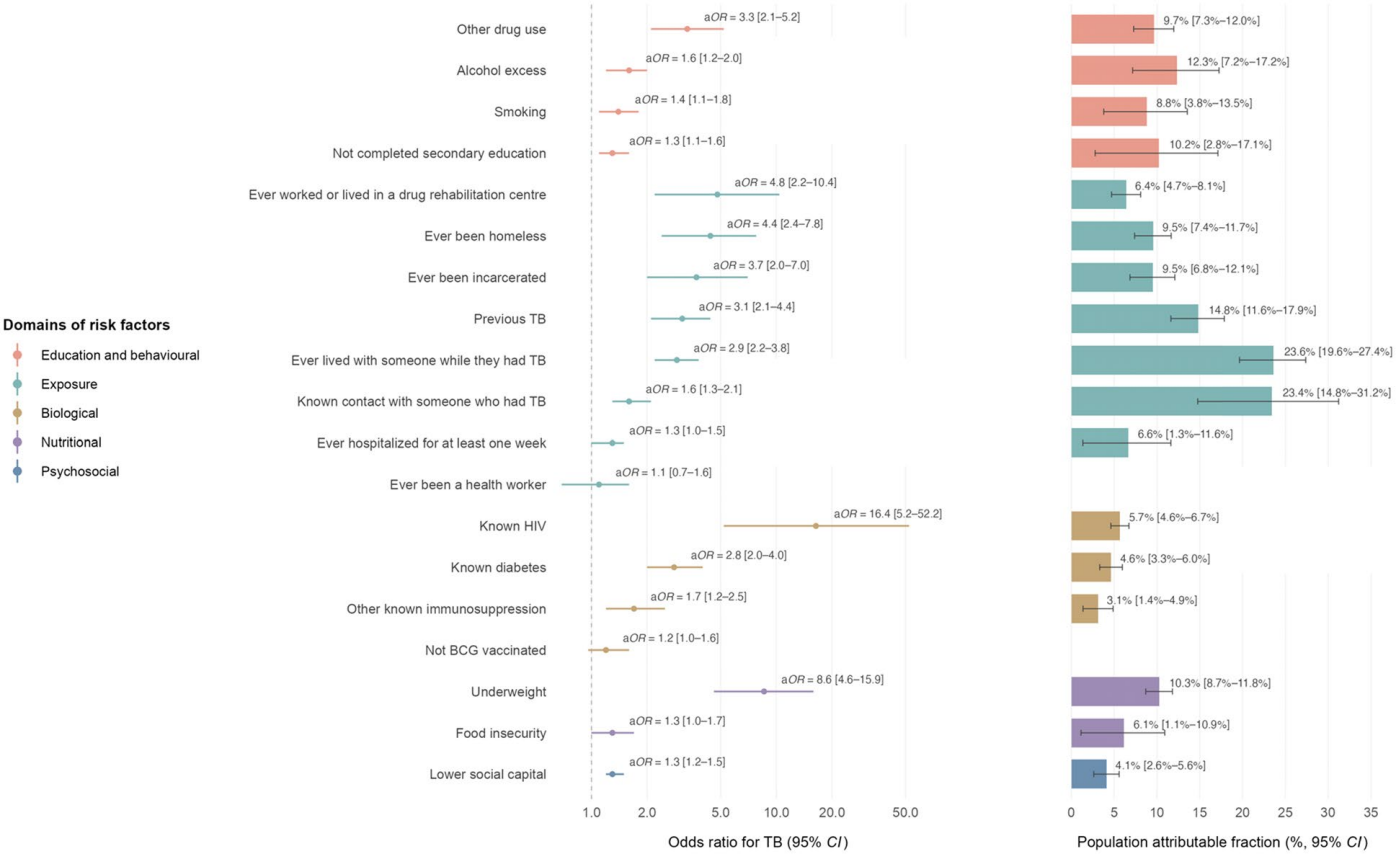
Associations between personal risk factors and tuberculosis (TB) (*n* = 3318). *BCG* Bacillus Calmette-Guérin. Odds ratios (a*OR*) and population attributable fractions (PAF) were adjusted for variables shown in the Supplementary Appendix, pages 6–8, based on the directed acyclic graph in Fig. 1. Error bars represent 95% confidence intervals (95% *CI*). For this analysis, social capital was considered as a continuous variable and the a*OR* represents odds of tuberculosis per standard deviation decrease in social capital score. The PAF represents a scenario where everyone in the population has the social capital of the average control participant. PAFs are not shown for ever being a health worker or BCG vaccination as the 95% *CI* crossed one

Personal risk factors for controls versus cases with tuberculosis are shown in Table 2, and their a*OR* for tuberculosis and PAF are shown in Fig. 4 and in the Supplementary Appendix pages 6–8. Notably, even after adjusting for household poverty and other demographic confounders, the PAF indicates that tuberculosis burden could be reduced by 10.2% (95% *CI:* 2.8–17.1) if all individuals complete secondary education. For the five key risk factors included in annual WHO reports, we estimated PAF for alcohol excess (PAF = 12.3%, 95% *CI:* 7.2–17.2); underweight (PAF = 10.3%, 95% *CI:* 8.7–11.8); smoking (PAF = 8.8%, 95% *CI:* 3.8–13.5); HIV (PAF = 5.7%, 95% *CI:* 4.6–6.7); and diabetes (PAF = 4.6%, 95% *CI:* 3.3–6.0). We also identified several other important risk factors including previous tuberculosis (PAF = 14.8%, 95% *CI:* 11.6–17.9); incarceration (PAF = 9.5%, 95% *CI:* 6.8–12.1); and lower social capital (PAF = 4.1%, 95% *CI:* 2.6–5.6).

**Table 2:** Personal risk factors for controls versus cases with tuberculosis (*n* = 3318)

		People without TB (controls, *n* = 981)	People with TB (cases, *n* = 2337)
Age (years)	Median (*IQR*)	38 (25–54)	31 (23–47)
Age group (years)	50 +	316 (32.8%)	512 (22.0%)
	30–49	300 (31.1%)	726 (31.2%)
	15–29	349 (36.2%)	1091 (46.8%)
Sex^1^	Female	592 (60.4%)	838 (35.9%)
	Male	389 (39.7%)	1499 (64.1%)
Place of birth	Lima	581 (59.2%)	1415 (61%)
	Province (e.g. mountains)	400 (40.8%)	920 (39.4%)
Education and behavioural risk factors			
Education	Completed secondary education	683 (69.8%)	1353 (58.0%)
	Not completed secondary education	296 (30.2%)	981 (42.0%)
Smoking	No	814 (83.2%)	1651 (70.9%)
	Yes	165 (16.9%)	679 (29.1%)
Alcohol excess	No	747 (83.0%)	1170 (68.0%)
	Yes	153 (17.0%)	551 (32.0%)
Other drug use	No	955 (97.7%)	2004 (86.2%)
	Yes	23 (2.4%)	322 (13.8%)
Exposure risk factors			
Previous TB	No	926 (94.5%)	1819 (78.0%)
	Yes	54 (5.6%)	514 (22.0%)
Known contact with someone who had TB	No	592 (63.2%)	802 (38.7%)
	Yes	345 (36.8%)	1271 (61.3%)
Ever lived with someone while they had TB	No	819 (84.4%)	1456 (64.1%)
	Yes	151 (15.6%)	816 (35.9%)
Ever hospitalized for at least one week	No	738 (75.5%)	1589 (68.6%)
	Yes	240 (24.5%)	729 (31.5%)
Ever been a health worker	No	922 (94.5%)	2250 (96.5%)
	Yes	54 (5.5%)	82 (3.5%)
Ever been incarcerated	No	964 (98.3%)	2028 (87.0%)
	Yes	17 (1.7%)	304 (13.0%)
Ever worked or lived in a drug rehabilitation centre	No	974 (99.3%)	2144 (91.9%)
	Yes	7 (0.7%)	189 (8.1%)
Ever been homeless	No	967 (98.7%)	2042 (87.6%)
	Yes	13 (1.3%)	289 (12.4%)
Biological risk factors			
BCG vaccination	Yes	843 (86.4%)	1945 (83.8%)
	No	133 (13.6%)	375 (16.2%)
Known diabetes	No	932 (95.2%)	2167 (92.8%)
	Yes	47 (4.8%)	168 (7.2%)
Known HIV	No	976 (99.7%)	2194 (94.0%)
	Yes	3 (0.3%)	141 (6.0%)
Other known immunosuppression	No	909 (93.7%)	2146 (92.7%)
	Yes	61 (6.3%)	170 (7.3%)
Nutritional risk factors			
BMI	Mean (*SD*)	26.5 (4.7)	22.7 (4.0)
Underweight	No	957 (98.9%)	2056 (88.4%)
	Yes	11 (1.1%)	270 (11.6%)
Number of days going to bed hungry in the last month because of lack of food	Mean (*SD*)	0.64 (2.1)	1.4 (3.7)
Food insecurity	No	837 (85.5%)	1762 (75.5%)
	Yes	142 (14.5%)	571 (24.5%)
Psychosocial risk factors			
Social capital	Mean score (*SD*)	0.00 (1.0)	−0.21 (0.61)

Data are *n* (%) unless otherwise stated. For percentages, the denominator is considered as individuals with data available

*IQR* interquartile range; *SD* standard deviation; *TB* tuberculosis; *BCG* Bacillus Calmette-Guérin; *BMI* body mass index

^1^Sex assigned at birth

### Social gradients in personal risk factors

**Fig. 5:**
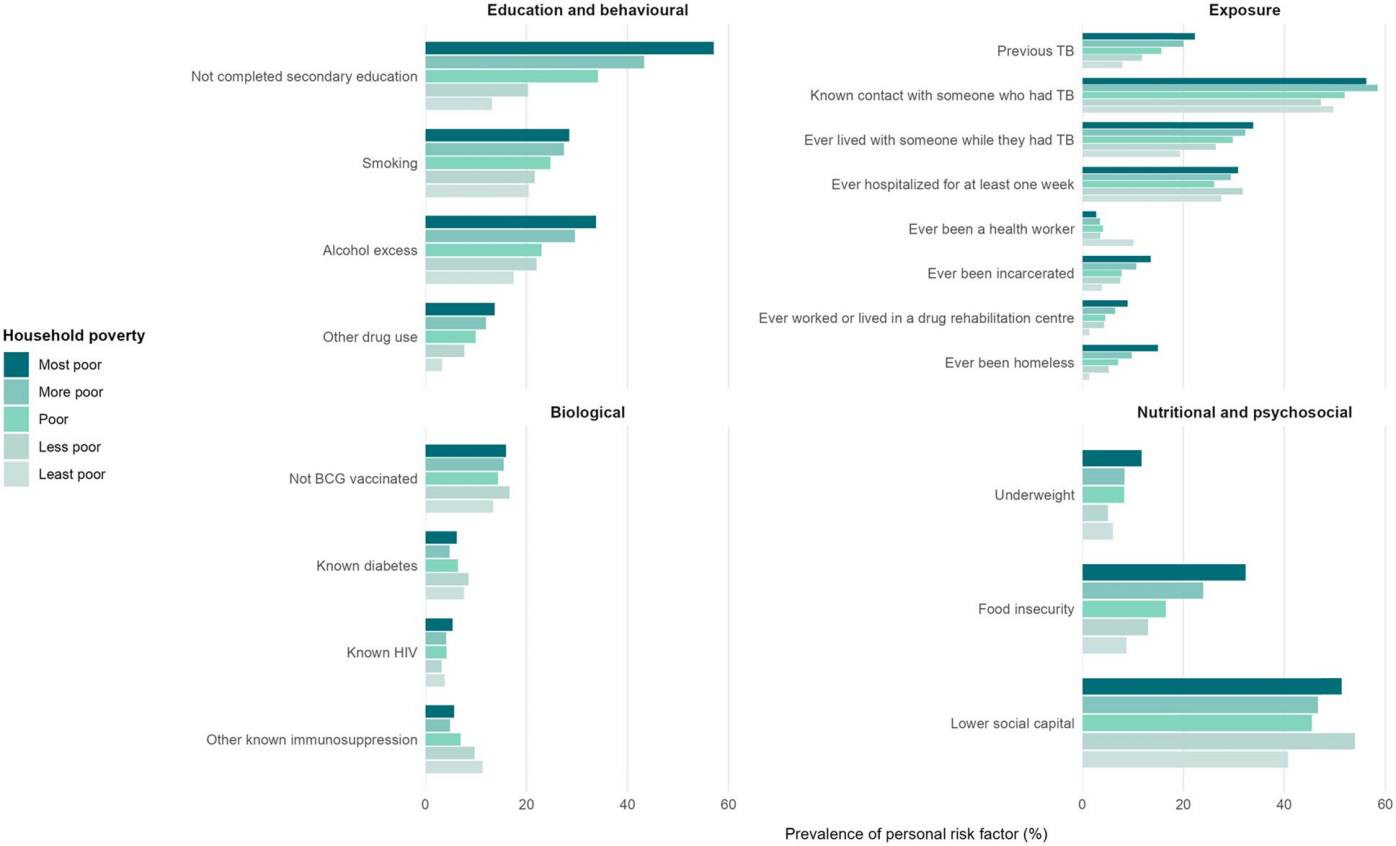
Social gradients in personal risk factors across quintiles of household poverty (*n* = 3318). *TB* tuberculosis. For this analysis, lower social capital was defined as less than the median social capital score. Numbers, percentages, and Cochran-Armitage test for linear trend p values across household poverty quintiles for each of these risk factors are shown in the Supplementary Appendix, pages 8-9

There were social gradients across quintiles of household poverty for the majority of these personal risk factors, which were more prevalent among people living in poorer households (Fig. 5 and Supplementary Appendix pages 8-9). These social gradients were particularly clear for education and substance misuse, tuberculosis exposures (e.g. incarceration and homelessness), and nutritional risk factors. Of note, HIV showed no social gradient, whilst diabetes (test for trend, *P* = 0.048) and other immunosuppression (test for trend, *P* < 0.001) were more prevalent among people living in less poor households.

## Discussion

This case–control study of over 3300 people provides rigorous evidence on the social determinants of tuberculosis in Peru, enhancing our understanding of tuberculosis epidemiology in this setting and informing the design of person-centred, equity-oriented interventions to reduce tuberculosis burden.

We demonstrated a clear association between household poverty and tuberculosis. Indeed, the social gradient in tuberculosis was so strong that approximately half of the tuberculosis in this setting could be prevented if the poorer half of households experience a reduction in poverty to the level of the less poor half. Importantly, because this benchmark is grounded in the actual living standards observed within the community and has already been achieved by half of the population, it represents a tangible, feasible target rather than a theoretical elimination of poverty. We also demonstrated that no single dimension of household poverty drives this association, with a household’s building quality and crowding (physical capital); human resources and skills (human capital); and finances and food spending (financial capital) all strongly and independently associated with tuberculosis. Notably, the exponential social gradient between household poverty and tuberculosis demonstrates that tuberculosis risk is especially pronounced among people living in extreme poverty.

These findings have important implications for global tuberculosis policy, where discourse around social determinants generally focusses on financial protection for people diagnosed with tuberculosis [26]. Although critically important, with evidence demonstrating these interventions are likely to improve treatment outcomes and reduce catastrophic costs [13, 27], tuberculosis-specific social protection for households already living with tuberculosis alone is unlikely to significantly contribute to reducing tuberculosis incidence. Our results suggest the emphasis of this discourse should shift to how poverty reduction and social protection interventions for communities and households with high social vulnerability indices can best prevent tuberculosis. This is supported by recent evidence from Brazil, which showed lower tuberculosis incidence and mortality among beneficiaries of a cash transfer programme targeted at poor families [28]. Importantly, whilst the dimension-specific PAF estimated in our study suggest that interventions targeting physical living conditions (e.g. housing improvements), financial resources (e.g. cash transfers), or human resources (e.g. education and labour programmes) could have similarly large effects, our findings suggest that interventions are likely to have more substantial, sustainable, and equitable impacts if they address the multidimensional aspects of poverty that increase tuberculosis risk. Placing poverty reduction and social protection at the centre of a local tuberculosis response requires generating new knowledge to understand what interventions are cost-effective and feasible locally; and investment, commitment, and collaboration from stakeholders at multiple levels. These include local community leaders and civil society organisations, healthcare workers and leaders, local and national government outside of the health system, and external funding agencies.

Notably, an individual’s education level remained associated with tuberculosis even after adjusting for household poverty, indicating that within poorer households, individuals with less education are at even higher risk of tuberculosis. This finding highlights the need to complement household-level interventions with targeted interventions addressing individual vulnerabilities. The other personal risk factors associated with tuberculosis and their PAF provide insight into these vulnerabilities and on how biosocial interventions, including social protection and tuberculosis active case finding and preventive treatment, may be designed to be person-centred, equity-oriented, and maximise impact. The PAF suggest interventions might have greater impact in this setting if they were expanded to people who have experienced substance misuse, homelessness, and incarceration in addition to the current focus on people with HIV and diabetes. Importantly, approximately one in seven cases were attributable to previous tuberculosis, showing that tuberculosis survivors constitute a priority group who should benefit from more intensive post-treatment interventions. Similarly, approximately one in four cases were attributable to ever having lived with someone who had tuberculosis, emphasising the importance of household contact investigation as a key intervention in the global tuberculosis response [29].

We also found strong associations between underweight, food insecurity and tuberculosis, highlighting the importance of improving nutrition to prevent tuberculosis. Although the prevalence of underweight among controls was low, it’s PAF was higher than many of the other risk factors we studied. If anything, we probably underestimated the importance of nutritional status because, whilst body mass index and tuberculosis incidence show a dose–response relationship [8], we used a strict, binary definition of underweight to be consistent with global estimates. Furthermore, our analyses would have been strengthened if we had data on the fuller spectrum of food insecurity experiences and diet [8]. We also found a clear association between lower social capital and tuberculosis, with a similar proportion of tuberculosis attributable to lower social capital as was to HIV and diabetes. The mechanisms through which social capital might protect against tuberculosis include improved informal care and support, information exchange, and providing a buffer against the negative health effects of poverty [30]. Finally, our results show that interventions targeted towards these personal risk factors would also enhance health equity, particularly those focussed on addressing substance misuse, tuberculosis exposures, and undernutrition. This is because nearly all were themselves highly socially determined and more prevalent among people living in poorer households.

Strengths of our study include the large sample size; the use of both discrete measures of household poverty and the derivation of composite multidimensional indices of household poverty; and our analytical approach, which was informed by a DAG to illustrate hypothesised causal relationships. Indeed, the major assumption underlying our conclusions is that the demonstrated associations are causal. Although we selected exposures because of a previously identified prospective association or because a plausible causal mechanism exists, we could not completely characterise temporality. This is particularly relevant for household poverty, underweight, and social capital because tuberculosis is impoverishing, and causes weight loss and social isolation. For underweight, although reverse causality could have resulted in overestimation, our results confirm our previous prospective research [31, 32] and our PAF estimate is lower than the WHO estimate for Peru [33]. For household poverty, we minimised reverse causality by deriving a multidimensional index, purposively including variables which are less sensitive to economic shocks. Importantly, any overestimation of the effect of poverty is likely to be countered because our case definition was based on people who were diagnosed with tuberculosis after accessing healthcare. Poorer people face greater barriers to diagnosis and are therefore more likely to have had a missed diagnosis during the study [34]. Relatedly, misclassification may have occurred if cases were diagnosed inappropriately with tuberculosis or if controls had undiagnosed tuberculosis, but this was minimised by linking symptomatic controls to healthcare and reclassifying them if they were diagnosed with tuberculosis. Recall and social desirability biases may have affected our results, e.g. if people underreported behaviours such as substance misuse. For HIV, all people with tuberculosis are offered an HIV test, so underdiagnosis in cases is likely to be very low. For controls, our estimate of HIV prevalence (0.3%) is similar to the World Bank estimate for Peru (0.4%) [35]. Of note, any underdiagnosis of comorbidities in controls would have had led us to overestimate the odds of tuberculosis for these exposures. Although the recruitment rate was high, the sex distribution of controls suggests some selection bias, which we hypothesise arose because men were more likely to be absent during daytime recruitment. Whilst this may have affected our ascertainment of personal risk factors, it should not have affected ascertainment of household poverty, and all analyses were adjusted for sex. Finally, although our results are only immediately generalisable to Peru, they have implications for other settings, especially the urban settings with a low HIV prevalence where most of the world’s tuberculosis occurs.

## Conclusions

Our results and other available evidence demonstrate the fundamental importance of prioritising an approach to tuberculosis that extends beyond biomedical solutions and the economic costs of tuberculosis to additionally focus on addressing the social determinants responsible for causing tuberculosis and entrenching inequity. Whilst there are challenges to achieving this in the short-term, the current global tuberculosis response is far from meeting its targets and radical changes are required. In the long-term, as well as being more equitable, reimagining our approach to tuberculosis by placing poverty reduction and social protection at its centre might increase efficiency and have greater, longer lasting impact for the world’s most vulnerable populations.

## Supplementary Information

Additional file 1.

## Data Availability

Deidentified participant data and a data dictionary will be made available on online repositories after publication of other studies undertaken as part of the PREVENT-TB study. In the interim, requests for data/analysis code can be made via the corresponding author.

## Abbreviations

WHO: World Health Organization
UN: United Nations
SDGs: Sustainable Development Goals
TB: Tuberculosis
MINSA: Ministry of Health
DAG: Directed acyclic graph
BMI: Body mass index
BCG: Bacillus Calmette-Guérin
SASCAT: Short adapted social capital assessment tool
SD: Standard deviation
IQR: Interquartile range
OR: Odds ratio
95% *CI*: 95% confidence interval
PAF: Population attributable fraction
PCA: Principal components analysis
