# Supplementary material for: The social determinants of tuberculosis: a case-control study characterising pathways to equitable intervention in Peru
Source: Infect Dis Poverty. 2025 Jun 20;14:53. doi: 10.1186/s40249-025-01324-6 (PMC12180230; doi:10.1186/s40249-025-01324-6)
Supplement: Supplementary file 1 — Additional file 1. [file 40249_2025_1324_MOESM1_ESM.docx]

The social determinants of tuberculosis:

a case-control study characterising pathways to equitable intervention

**Supplementary appendix**

**Directed acyclic graph**

We conceptualised household poverty as a time-stable exposure, which could be influenced by an individual’s age, sex, community, and place of birth. We then hypothesised that household poverty (and age, sex, community, and place of birth) influenced the education level of the individuals living in that household, and their exposure to key domains of personal risk factors. We also hypothesised that these personal risk factors had some hierarchy between them. Behavioural risk factors (e.g. smoking, alcohol excess, drug use) were hypothesised to influence an individual’s risk of biological risk factors (e.g. HIV, diabetes), exposure risk factors (e.g. previous incarceration, known tuberculosis contact), nutritional risk factors (current body mass index), and psychosocial risk factors (social capital). Biological risk factors were hypothesised to influence exposure risk factors (e.g. previous hospitalisation, previous tuberculosis), nutritional risk factors (current body mass index), and psychosocial risk factors (social capital). Exposure risk factors (e.g. previous incarceration, ever having lived or worked in a drug rehabilitation centre) were hypothesised to influence psychosocial risk factors (social capital), and previous tuberculosis was also hypothesised to influence nutritional risk factors (food security and current body mass index). Within the exposure risk factors, we also hypothesised a hierarchy (e.g. previous incarceration influencing known tuberculosis contact and previous tuberculosis; and known tuberculosis contact influencing previous tuberculosis); and within the nutritional risk factors we hypothesised that food security influenced current body mass index. We could not allow for the possibility that these personal risk factors could themselves influence household poverty (e.g. previous tuberculosis causing impoverishment) because acyclic graphs only allow for unidirectional relationships, and this would have required measurements at multiple time points, which were not available.

**Missing data**

For the great majority of variables, the proportion of participants with missing data was very small (<1%) (Tables S1 and S2). Multiple imputation with chained equations was used to replace missing values assuming the data were missing at random (i.e. that the probability a value was missing was not dependent on unobserved data). Imputation was undertaken in two steps. First, missing values for household poverty variables were imputed including all household poverty variables in the predictor equations. Then, after a composite household poverty index was derived, missing values for personal risk factors were imputed including all the personal risk factor variables and the household poverty index in the predictor equations. This was done for efficiency reasons so that instead of including all 27 household poverty variables in the predictor equations for personal risk factors, only one (household poverty) was included. Imputation equations additionally included age, sex, community, and case status. Ten imputations were generated, and Rubin’s rules were used to combine estimates for the analysis across the imputed datasets.

**Table S1.** Missing data for household poverty variables (n=2,713)

| **Variable** | **Number with data missing (%)** |
| --- | --- |
| Crowding | 60 (2.2%) |
| Home ownership | 11 (0.41%) |
| Wall quality | 3 (0.11%) |
| Floor quality | 7 (0.26%) |
| Water supply | 6 (0.22%) |
| Toilet | 6 (0.22%) |
| Electricity | 5 (0.18%) |
| Cooking fuel | 178 (6.6%) |
| Television ownership | 13 (0.48%) |
| Fridge ownership | 4 (0.15%) |
| Iron ownership | 19 (0.70%) |
| Stove ownership | 5 (0.18%) |
| Mobile phone | 7 (0.26%) |
| Landline | 4 (0.15%) |
| Radio | 6 (0.22%) |
| Coffee maker | 13 (0.48%) |
| Wardrobe | 7 (0.26%) |
| Food processor ownership | 14 (0.52%) |
| Female head of household education | 141 (5.2%) |
| Male head of household education | 123 (4.5%) |
| Used internet in last week | 26 (0.96%) |
| Number of days of food available | 25 (0.92%) |
| Food spending per person | 74 (2.7%) |
| Household income per person | 371 (14%) |
| Any savings | 197 (7.3%) |
| Any debt | 174 (6.4%) |
| Bank account | 502 (19%) |

**Table S2.** Missing data for personal risk factors (n=3,318)

| **Variable** | **Number with data missing (%)** |
| --- | --- |
| Age | 24 (0.7%) |
| Sex | 0 (0.0%) |
| Place of birth | 2 (0.06%) |
| Previous TB | 5 (0.15%) |
| Known contact with someone who had tuberculosis | 308 (9.3%) |
| Ever lived with someone while they had tuberculosis | 76 (2.3%) |
| Ever hospitalized for at least 1 week | 22 (0.66%) |
| Ever been a health worker | 10 (0.30%) |
| Ever been incarcerated | 5 (0.15%) |
| Ever worked or lived in a drug rehabilitation centre | 4 (0.12%) |
| Ever been homeless | 7 (0.21%) |
| BCG vaccination | 22 (0.66%) |
| Known diabetes | 4 (0.12%) |
| Known HIV | 4 (0.12%) |
| Other known immunosuppression | 32 (0.96%) |
| Body mass index | 24 (0.72%) |
| Days hungry and food insecurity | 6 (0.18%) |
| Smoking | 9 (0.27%) |
| Alcohol excess | 697 (21%) |
| Other drug use | 14 (0.42%) |
| Education | 5 (0.15%) |
| Social capital | 71 (2.1%) |

Table S3. Associations between household poverty and tuberculosis (n=3,318).

|  |  |  | **Controls (n=981)** | **Cases (n=2,337)** | **aOR***  **(95% CI)** | **p value** | **PAF (95%CI)** |
| --- | --- | --- | --- | --- | --- | --- | --- |
| **Discrete measures of household poverty** | Crowding (n=3,245) | <1 person per room | 119 (12%) | 234 (10%) | Reference | Reference | 8.8% (4.7-13) |
|  |  | 1 to < 2 people per room | 556 (58%) | 1,112 (49%) | 1.0 (0.71-1.5) | 0.88 |  |
|  |  | 2 to < 3 people per room | 214 (22%) | 570 (25%) | 1.1 (0.72-1.8) | 0.58 |  |
|  |  | 3 to < 4 people per room | 56 (5.8%) | 175 (7.7%) | 1.5 (0.75-2.9) | 0.26 |  |
|  |  | 4 or more people per room | 14 (1.5%) | 195 (8.5%) | 3.9 (1.6-9.6) | 0.003 |  |
|  | Female head of the household education (n=3,167) | University educated | 150 (16%) | 178 (8.1%) | Reference | Reference | 26% (17-34) |
|  |  | Completed secondary education | 414 (43%) | 714 (23%) | 1.2 (0.79-1.7) | 0.43 |  |
|  |  | Incomplete secondary education | 314 (33%) | 882 (40%) | 1.7 (1.1-2.6) | 0.009 |  |
|  |  | No female head of the household | 87 (9.0%) | 428 (19%) | 2.9 (1.7-4.8) | <0.001 |  |
|  | Household income per person (n=2,613) | Above the national poverty line | 265 (61%) | 998 (46%) | Reference | Reference | 28% (19-35) |
|  |  | Below the national poverty line | 173 (39%) | 1,177 (54%) | 2.0 (1.5-2.7) | <0.001 |  |
| **Principal component analysis derived measures of household poverty** | Overall household poverty (n=3,318) | Less poor | 525 (54%) | 715 (31%) | Reference | Reference | 47% (40-54) |
|  |  | Poorer | 456 (46%) | 1,622 (69%) | 3.1 (2.3-4.2) | <0.001 |  |
|  | Physical capital (n=3,318) | Less poor | 513 (52%) | 804 (34%) | Reference | Reference | 31% (20-41) |
|  |  | Poorer | 468 (48%) | 1,533 (66%) | 1.9 (1.4-2.5) | <0.001 |  |
|  | Human capital (n=3,318) | Less poor | 568 (58%) | 941 (40%) | Reference | Reference | 26% (16-35) |
|  |  | Poorer | 413 (42%) | 1,396 (60%) | 1.8 (1.4-2.3) | <0.001 |  |
|  | Financial capital (n=3,318) | Less poor | 456 (46%) | 782 (33%) | Reference | Reference | 28% (17-38) |
|  |  | Poorer | 525 (54%) | 1,555 (67%) | 1.7 (1.3-2.3) | <0.001 |  |

aOR=Adjusted odds ratio for tuberculosis; PAF=Population attributable fraction of tuberculosis; 95%CI=95% confidence interval

*aOR and thus PAF were adjusted for age, sex, community, and place of birth for all variables shown here. For discrete measures of household poverty, aOR/PAF were adjusted for the other variables in the table. For physical, human, and financial capital, aOR/PAF were adjusted for the other dimensions.

**Table S4.** Social gradient in tuberculosis (TB) across deciles of household poverty (n=3,318)

| **Household poverty decile** | **Controls (n=981)** | **Cases (n=2,337)** | **aOR* (95%CI)** | **p value** |
| --- | --- | --- | --- | --- |
| 1 (poorest) | 100 (10%) | 492 (21%) | 12.6 (6.9-23.2) | <0.001 |
| 2 | 99 (10%) | 415 (18%) | 10.5 (5.6-19.7) | <0.001 |
| 3 | 96 (9.8%) | 349 (15%) | 9.1 (5.0-16.8) | <0.001 |
| 4 | 101 (10%) | 274 (12%) | 6.0 (3.2-11.1) | <0.001 |
| 5 | 97 (9.9%) | 193 (8.3%) | 3.9 (2.1-7.1) | <0.001 |
| 6 | 97 (9.9%) | 113 (4.8%) | 2.1 (1.1-4.0) | 0.029 |
| 7 | 98 (10%) | 168 (7.2%) | 3.8 (2.1-6.9) | <0.001 |
| 8 | 98 (10%) | 153 (6.6%) | 2.7 (1.5-5.0) | 0.001 |
| 9 | 97 (9.9%) | 136 (5.8%) | 2.6 (1.4-4.9) | 0.002 |
| 10 (least poor) | 98 (10%) | 54 (2.3%) | Reference | Reference |

aOR=Adjusted odds ratio for tuberculosis; 95%CI=95% confidence interval

*OR were adjusted for age, sex, community, and place of birth.

**Table S5**. Associations between personal risk factors and tuberculosis (n=3,318)

|  |  | **aOR**  **(95% CI)** | **p value** | **PAF (95%CI)** | **Adjustment set for calculating aOR/PAF** |
| --- | --- | --- | --- | --- | --- |
| **Education and behavioural** | | | | | |
| Education | Completed secondary education | Reference | Reference | 10.2% (2.8-17.1) | Age, sex, community, household poverty, place of birth |
|  | Not completed secondary education | 1.3 (1.1-1.6) | 0.013 |  |  |
| Smoking | No | Reference | Reference | 8.8% (3.8-13.5) | Age, sex, community, education, household poverty, place of birth |
|  | Yes | 1.4 (1.1-1.8) | 0.002 |  |  |
| Alcohol excess | No | Reference | Reference | 12.3% (7.2-17.2) | Age, sex, community, education, household poverty, place of birth |
|  | Yes | 1.6 (1.2-2.0) | <0.001 |  |  |
| Other drug use (e.g. cocaine) | No | Reference | Reference | 9.7% (7.3-12.0) | Age, sex, community, education, household poverty, place of birth |
|  | Yes | 3.3 (2.1-5.2) | <0.001 |  |  |
| **Exposure** | | | | | |
| Previous TB | No | Reference | Reference | 14.8% (11.6-17.9) | Age, sex, community, education, household poverty, place of birth, all exposure risk factors, all biological risk factors, all behavioural risk factors. |
|  | Yes | 3.1 (2.1-4.4) | <0.001 |  |  |
| Known contact with someone who had TB | No | Reference | Reference | 23.4% (14.8-31.2) | Age, sex, community, education, household poverty, place of birth, all exposure risk factors except previous TB, all behavioural risk factors |
|  | Yes | 1.6 (1.3-2.1) | <0.001 |  |  |
| Ever lived with someone while they had TB | No | Reference | Reference | 23.6% (19.6-27.4) | Age, sex, community, education, household poverty, place of birth |
|  | Yes | 2.9 (2.2-3.8) | <0.001 |  |  |
| Ever hospitalized for at least one week | No | Reference | Reference | 6.6% (1.3-11.6) | Age, sex, community, education, household poverty, place of birth, all biological risk factors (except BCG), all behavioural risk factors |
|  | Yes | 1.3 (1.0-1.5) | 0.02 |  |  |
| Ever been a health worker | No | Reference | Reference | NA | Age, sex, community, education, household poverty, place of birth |
|  | Yes | 1.1 (0.69-1.6) | 0.78 |  |  |
| Ever been incarcerated | No | Reference | Reference | 9.5% (6.8-12.1) | Age, sex, community, education, household poverty, place of birth, all behavioural risk factors |
|  | Yes | 3.7 (2.0-7.0) | <0.001 |  |  |
| Ever worked or lived in a drug rehabilitation centre | No | Reference | Reference | 6.4% (4.7-8.1) | Age, sex, community, education, household poverty, place of birth, all behavioural risk factors |
|  | Yes | 4.8 (2.2-10.4) | <0.001 |  |  |
| Ever been homeless | No | Reference | Reference | 9.5% (7.4-11.7) | Age, sex, community, education, household poverty, place of birth, all behavioural risk factors |
|  | Yes | 4.4 (2.4-7.8) | <0.001 |  |  |
| **Biological** | | | | | |
| BCG vaccination | Yes | Reference | Reference | NA | Age, sex, community, education, household poverty, place of birth |
|  | No | 1.2 (0.96-1.6) | 0.102 |  |  |
| Known diabetes | No | Reference | Reference | 4.6% (3.3-6.0) | Age, sex, community, education, household poverty, place of birth, all behavioural risk factors |
|  | Yes | 2.8 (2.0-4.0) | <0.001 |  |  |
| Known HIV | No | Reference | Reference | 5.7% (4.6-6.7) | Age, sex, community, education, household poverty, place of birth, all behavioural risk factors |
|  | Yes | 16.4 (5.2-52.2) | <0.001 |  |  |
| Other known immunosuppression | No | Reference | Reference | 3.1% (1.4-4.9) | Age, sex, community, education, household poverty, place of birth, all behavioural risk factors |
|  | Yes | 1.7 (1.2-2.5) | 0.002 |  |  |
| **Nutritional** | | | | | |
| Underweight | No | Reference | Reference | 10.3% (8.7-11.8) | Age, sex, community, education, household poverty, place of birth, previous TB, all biological risk factors (except BCG), food insecurity, all behavioural risk factors |
|  | Yes | 8.6 (4.6-15.9) | <0.001 |  |  |
| Food insecurity | No | Reference | Reference | 6.1% (1.1-10.9) | Age, sex, community, education, household poverty, place of birth, previous TB |
|  | Yes | 1.3 (1.0-1.7) | 0.028 |  |  |
| **Psychosocial** | | | | | |
| Lower social capital | per SD decrease in score | 1.3 (1.2-1.5) | <0.001 | 4.1% (2.6-5.6) | Age, sex, community, education, household poverty, place of birth, previous TB, previous incarceration, ever worked or lived in a drug rehabilitation centre, all behavioural risk factors, all biological risk factors (except BCG) |

TB=tuberculosis; BCG=Bacillus Calmette-Guérin; NA=Not applicable; aOR=Adjusted odds ratio for tuberculosis; PAF=Population attributable fraction; 95%CI=95% confidence interval

**Table S6.** Social gradients in personal risk factors across quintiles of household poverty (n=3,318)

| **Domain** | **Personal risk factor** | **Cochran-Armitage test for linear trend** | **Least poor** | **Less poor** | **Poor** | **More poor** | **Poorest** |
| --- | --- | --- | --- | --- | --- | --- | --- |
| Education and behavioural | Not completed secondary education (n=3,313) | <0.001 | 13% | 20% | 34% | 43% | 57% |
| Education and behavioural | Smoking (n=3,309) | <0.001 | 20% | 22% | 25% | 27% | 29% |
| Education and behavioural | Alcohol excess (n=2,621) | <0.001 | 17% | 22% | 23% | 30% | 34% |
| Education and behavioural | Other drug use (n=3,304) | <0.001 | 3.3% | 7.7% | 10% | 12% | 14% |
| Exposure | Previous TB (n=3,313) | <0.001 | 8% | 12% | 16% | 20% | 22% |
| Exposure | Known contact with someone who had TB (n=3,010) | <0.001 | 50% | 47% | 52% | 58% | 56% |
| Exposure | Ever lived with someone while they had TB (3,242) | <0.001 | 19% | 26% | 30% | 32% | 34% |
| Exposure | Ever hospitalized for at least one week (n=3,296) | 0.42 | 28% | 32% | 26% | 29% | 31% |
| Exposure | Ever been a health worker (n=3,308) | <0.001 | 10% | 3.6% | 4.1% | 3.5% | 2.7% |
| Exposure | Ever been incarcerated (n=3,313) | <0.001 | 3.8% | 7.5% | 7.7% | 11% | 14% |
| Exposure | Ever worked or lived in a drug rehabilitation centre (n=3,314) | <0.001 | 1.4% | 4.3% | 4.5% | 6.5% | 8.9% |
| Exposure | Ever been homeless (n=3,311) | <0.001 | 1.4% | 5.2% | 7.1% | 10% | 15% |
| Biological | Not BCG vaccinated (n=3,296) | 0.48 | 13% | 17% | 14% | 16% | 16% |
| Biological | Known diabetes (n=3,314) | 0.048 | 7.7% | 8.6% | 6.4% | 4.8% | 6.2% |
| Biological | Known HIV (n=3,314) | 0.058 | 3.8% | 3.2% | 4.2% | 4.2% | 5.4% |
| Biological | Other known immunosuppression (n=3,286) | <0.001 | 11% | 10% | 7.0% | 4.9% | 5.7% |
| Nutritional | Underweight (n=3,294) | <0.001 | 6.1% | 5.0% | 8.3% | 8.3% | 12% |
| Nutritional | Food insecurity (n=3,312) | <0.001 | 8.7% | 13% | 17% | 24% | 32% |
| Psychosocial | Lower social capital (n=3,247) | 0.084 | 41% | 54% | 45% | 47% | 51% |

TB=tuberculosis; BCG=Bacillus Calmette-Guérin

White cell highlighting indicates risk factors demonstrating a social gradient, being more prevalent among people living in poorer households. Blue cell highlighting indicates risk factors demonstrating an inverse social gradient, being more prevalent among people living in less poor households. Grey cell highlighting indicates risk factors not showing any social gradient. For this analysis, lower social capital was defined as less than the median social capital score.
